# Development and Application of an In Vitro Method to Evaluate Anthracnose Resistance in Soybean Germplasm

**DOI:** 10.3390/plants11050657

**Published:** 2022-02-28

**Authors:** Longming Zhu, Lele Feng, Xiaomin Yu, Xujun Fu, Qinghua Yang, Hangxia Jin, Fengjie Yuan

**Affiliations:** 1 Institute of Crop and Nuclear Technology Utilization, Zhejiang Academy of Agricultural Sciences, Hangzhou 310021, China; zlmsllzly@163.com (L.Z.); yuxm@zaas.ac.cn (X.Y.); xujunfu@hotmail.com (X.F.); tsingyang2009@163.com (Q.Y.); jinhangxia@126.com (H.J.); 2 College of Plant Science and Technology, Beijing University of Agriculture, Beijing 102206, China; waterlily27@126.com

**Keywords:** anthracnose, *Colletotrichum truncatum*, resistance evaluation, soybean

## Abstract

Anthracnose caused by *Colletotrichum truncatum* is a major fungal disease of soybean, especially vegetable soybean (edamame). Studies of this disease have mainly focused on resistance evaluation, but the primary methods used—in vivo inoculation of pods or plants under greenhouse or field conditions—have limitations with respect to accuracy, stability, scale, and environmental safety. In this study, we developed a method for inoculating pods in vitro by soaking in a mycelial suspension. We optimized the crucial components, including the mycelial suspension concentration (40 to 60 mg mL^−1^), the maturity of the sampled pods (15 days after flowering), and the post-inoculation incubation period (5 days). Application of the mycelial suspension by soaking rather than spraying improved the efficiency of inoculation and made large-scale evaluation possible. Using this method, we evaluated 589 soybean germplasm resources (275 cultivars, 233 landraces, and 81 wild accessions). We identified 25 highly resistant cultivars, 11 highly resistant landraces, but only one highly resistant wild accession. Our results will aid future research on soybean anthracnose resistance, including gene discovery, the elucidation of molecular mechanisms, and the breeding of resistant cultivars.

## 1. Introduction

Anthracnose, caused by *Colletotrichum truncatum*, is a major fungal disease of soybean (*Glycine max* (L.) Merrill) [1]. All the aboveground parts of soybean plants can be infected at any stage of development by *C. truncatum*. During the early stages of infection, typical distinctive symptoms of anthracnose are irregularly shaped brown blotches on cotyledons, stems, petioles, leaves, and pods, which gradually develop into dark, depressed, and irregular lesions. As the disease progresses, leaf rolling, premature defoliation, necrosis of laminar veins and pods, and seed abortion may be observed. Moreover, infected seeds often become discolored and can undergo pre- or post-emergence damping off [2,3,4,5,6].

Soybean anthracnose is more prevalent in subtropical and tropical regions, such as the southern growing areas of China and the USA, as well as those of northern Argentina, Brazil, Thailand, and India [2]. Estimated yield losses attributable to anthracnose are significant. In the top eight soybean-producing countries (Argentina, Bolivia, Brazil, Canada, China, India, Paraguay, and the USA), grain-yield losses of 25.4 million tons have been reported, including a record 16.6 million tons in China in 2006 [7]. Anthracnose is an especially serious problem in vegetable soybean (edamame) production. As fresh pods are the final product, their commodity value is greatly reduced as soon as disease blotches or lesions develop. The resulting economic loss per unit area is higher than that experienced in traditional soybean production. China is the largest producer and exporter of vegetable soybean worldwide, with a cultivated area of 1.0–1.5×10^7^ ha and pod yields of 5.0×10^5^ tons per year [8]. However, vegetable soybean production in China is concentrated in the southeastern coastal area, where anthracnose frequently occurs because of the warm, humid climate. For example, in Zhejiang Province, the anthracnose incidence of soybean plants is about 50% and that of soybean pods is about 30% in typical fields; in severely infected fields, the incidence can increase to >90% of soybean plants and >50% of soybean pods [9,10]. 

Current methods used to manage the disease and limit pathogen spread during soybean production include rapid diagnosis, biological control, planting of pathogen-free seeds, and chemical control [5,11,12,13]. These methods are economically costly and/or ecologically unsound; in the case of vegetable soybean, food safety is also a concern. The use of disease-resistant cultivars is, therefore, the best option, especially for vegetable soybean production. However, the mainly cultivated vegetable soybean cultivars and breeding germplasm resources currently available in China, are not resistant to soybean anthracnose. Therefore, screening, identifying resistance resources, and breeding high-yield and resistant new cultivars are urgently needed for vegetable soybean production. 

Screening of soybean germplasm for anthracnose resistance is a crucial step in the breeding of resistant cultivars. To date, researchers have evaluated the anthracnose resistance of modern soybean cultivars from the USA, China, Brazil, and India. However, the resistance evaluation scale was small and mainly focused on cultivars; only a few resistant sources have been identified, and no immune ones have been reported [3,14,15,16].

Methods used to evaluate anthracnose resistance have differed in several respects, including the means of disease introduction (natural disease and artificial inoculation), the objects of evaluation (leaves, pods, and seedling), the inoculum type (conidia and mycelia), the incubation conditions (greenhouse and field), and the disease severity scoring (visual assessment of inoculated plants and pods) [14,16,17,18,19,20,21]. However, these methods have all been in vivo, which has limitations. For example, several years of repetition are required to obtain accurate results; thus, the outcome is strongly influenced by environmental factors. The establishment of a dedicated area, which is time-consuming and labor-intensive, may be required to avoid affecting other planting schedules. Furthermore, large-scale resistance evaluation is challenging.

In this study, we developed a rapid, reliable, and precise method for the evaluation of soybean anthracnose resistance. This method was used to evaluate the anthracnose resistance of various Chinese soybean germplasm resources, including cultivars, landraces, and wild accessions, and identified sources with superior resistance collected from across the Huanghuai region and the region south of the Yangtze River [22]. Our findings provide a foundation for the discovery of resistance genes, the elucidation of their associated molecular mechanisms, and the breeding of resistant cultivars.

## 2. Results

### 2.1. Determination of the Optimal Mycelial Suspension Concentration

**Figure 1:**
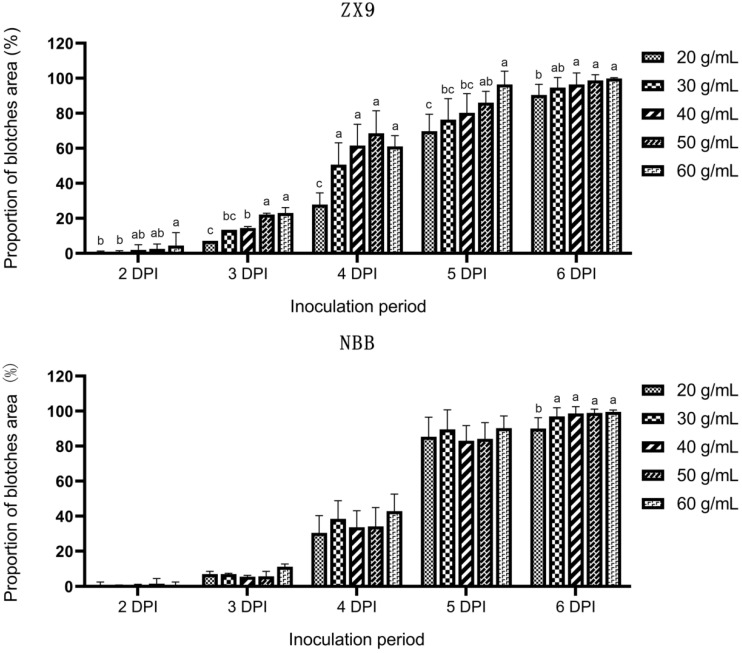
Proportion of the pod surface area covered with blotches 2 to 6 days after inoculation of the pods of Zhexian No. 9 (ZX9) and Nanhua Black Bean (NBB) soybean, with different concentrations of mycelial suspensions. The different letters above the columns indicate significant differences (*p* = 0.05) determined by two-tailed *t*-tests.

Compared with conidial suspensions, mycelial suspensions are more infectious and easier to prepare [14]. For simplicity, and to maximize disease pressure, we therefore, used a mycelial suspension as the inoculum in this study. As shown in Figure 1, the severity of anthracnose disease on the pods of Zhexian No.9 (ZX9) soybeans increased slightly as the mycelial suspension concentration was increased. No significant differences were observed within the range of 30 to 50 mg mL^−^^1^ over the entire incubation period, except at 3 days post-inoculation (DPI). In the cultivar Nanhua Black Bean (NBB), increasing the concentration of the mycelial suspension had no significant effect on disease severity over the entire incubation period. The only significant differences were observed between 20 and 60 mg mL^−^^1^ at 6 DPI (Figure 1). 

Our results indicate that a concentration of 30 to 50 mg mL^−^^1^ is sufficient to ensure infection with no significant effect on disease severity. This conclusion is consistent with the findings of a previous study [14]. The concentration of the mycelial suspension sufficient to cause full infection of the soybean plants or pods does not significantly affect anthracnose disease severity. Therefore, any mycelial suspension concentration within the appropriate range can be used.

### 2.2. Determination of the Optimal Maturity Stage for Sampling Pods

**Figure 2:**
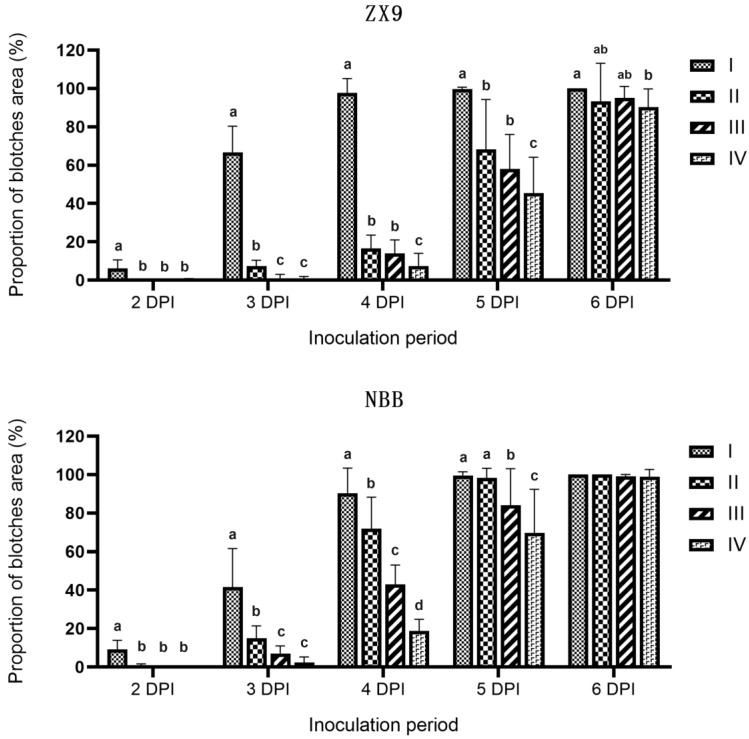
Proportion of the pod surface area covered with blotches 2 to 6 days after inoculation, of the pods at different stages of maturity, of Zhexian No. 9 (ZX9) and Nanhua Black Bean (NBB) soybean, inoculated with mycelial suspension. I, II, III, and IV represent four different stages of maturity, ordered from youngest to oldest. The different letters above the columns indicate significant differences (*p* = 0.05) determined by two-tailed *t*-tests.

In contrast to the previously reported in vivo inoculation methods [14,16,17,18,19,20,21], we used an in vitro pod inoculation approach. To determine the optimal maturity stage for sampling pods, we examined the relationship between pod maturity and disease severity. Four different stages of maturity were examined: type-I ca. 5 days after flowering (DAF), when the pods were small and soft; type-II ca. 15 DAF, when the pods were fully grown and no longer soft but still flat because the seeds had not yet begun to protrude; type-III ca. 25 DAF, when the pods were half filled; and type-IV ca. 40 DAF, when the pods were completely filled, which is when vegetable soybean is harvested. We observed significant differences in disease severity between pods of different maturities, with the most rapid disease progression occurring in younger pods, especially type-I pods. At 2 DPI, obvious blotches were observed on type-I pods, whereas pods at other stages of maturity had only a few small blotches. At 4 DPI, almost 100% of the surface of type-I pods was covered with blotches; in contrast, older pods did not reach the same state until 6 DPI (Figure 2). Choosing young pods can save incubation time and improve efficiency, making it more conducive for the screening of superior disease-resistant germplasm resources. However, the type-I pods were too young, and disease development was too rapid; disease development must be accurate and predictable for experimentation, otherwise, large-scale resistance evaluation would be prone to error. Type-II pods met the requirements and were selected for sampling and inoculation. 

### 2.3. Determination of the Optimal Post-Inoculation Incubation Time

**Figure 3:**
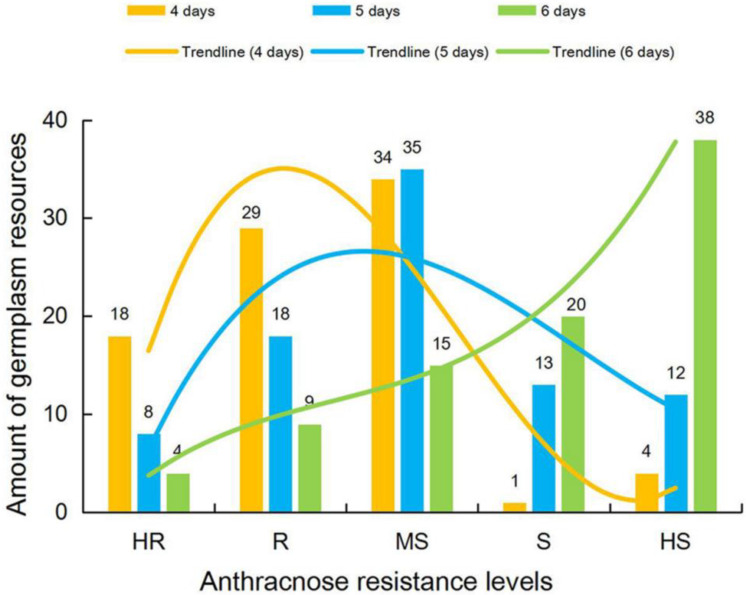
Number of soybean accessions at each level of soybean anthracnose resistance, at three different post-inoculation incubation times. HR, highly resistant; R, resistant; MS, moderately susceptible; S, susceptible; HS, highly susceptible.

None of the 86 tested germplasm resources exhibited immunity to soybean anthracnose. At 4 DPI, most accessions (44; 51.16%) were classified as resistant (16 highly resistant (HR) and 28 resistant (R)). At 6 DPI, susceptible germplasm resources (58; 67.44%) predominated, with 20 being classified as highly susceptible (HS) and 38 as susceptible (S). At 5 DPI, the distribution of resistance levels was reasonable and close to a normal distribution (Figure 3). We, therefore, considered a 5 day incubation time to be optimal.

### 2.4. Comparison of Inoculation by Spraying vs. Soaking

During our evaluation of the optimal post-inoculation incubation time, we realized that spray-based inoculation was time-consuming. Thus, to improve inoculation efficiency, we investigated the utility of inoculation by soaking.

**Figure 4:**
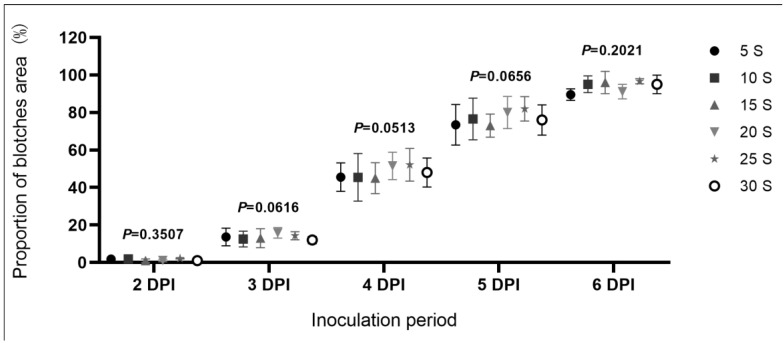
Proportion of the pod surface area covered by blotches 2 to 6 days after the inoculation of the pods of Zhexian No. 9 (ZX9) soybean with mycelial suspensions, administered by soaking for 5, 10, 15, 20, 25, and 30 s.

**Figure 5:**
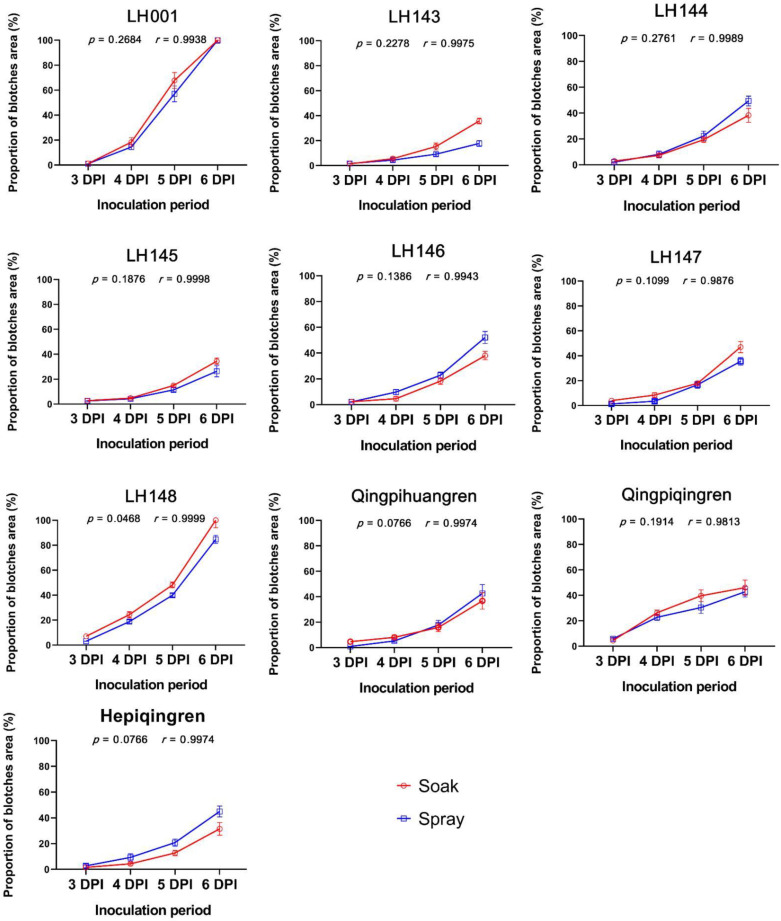
Proportion of the pod surface area covered by blotches 3 to 6 days after inoculation of the pods from 10 soybean accessions with the mycelial suspensions, administered by soaking or spraying. In each plot, the correlation coefficient (*r*) and the significance of differences (*p*-value) between the two methods are shown for each accession.

We first tested the effect of soaking duration on disease severity. Soaking for 5 s appeared to be sufficient to fully inoculate the pods and increasing the soaking period from 5 s to 30 s had no significant effect on disease severity (Figure 4). When we compared inoculation by spraying and soaking, Pearson correlation coefficients (*r*-values) were high and all *p*-values were greater than 0.05, indicating that disease progression and severity were consistent between the two methods (Figure 5). Inoculation by soaking can, therefore, be used instead of spray inoculation to improve inoculation efficiency and facilitate large-scale evaluation of anthracnose resistance.

### 2.5. Evaluation of Soybean Germplasm Resources for Anthracnose Resistance

**Figure 6:**
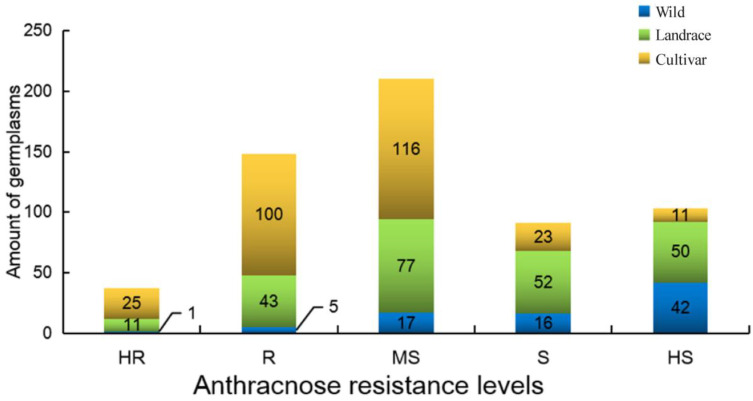
Distribution of the 589 soybean germplasm accessions according to the level of soybean anthracnose resistance. HR, highly resistant; R, resistant; MS, moderately susceptible; S, susceptible; HS, highly susceptible.

**Figure 7:**
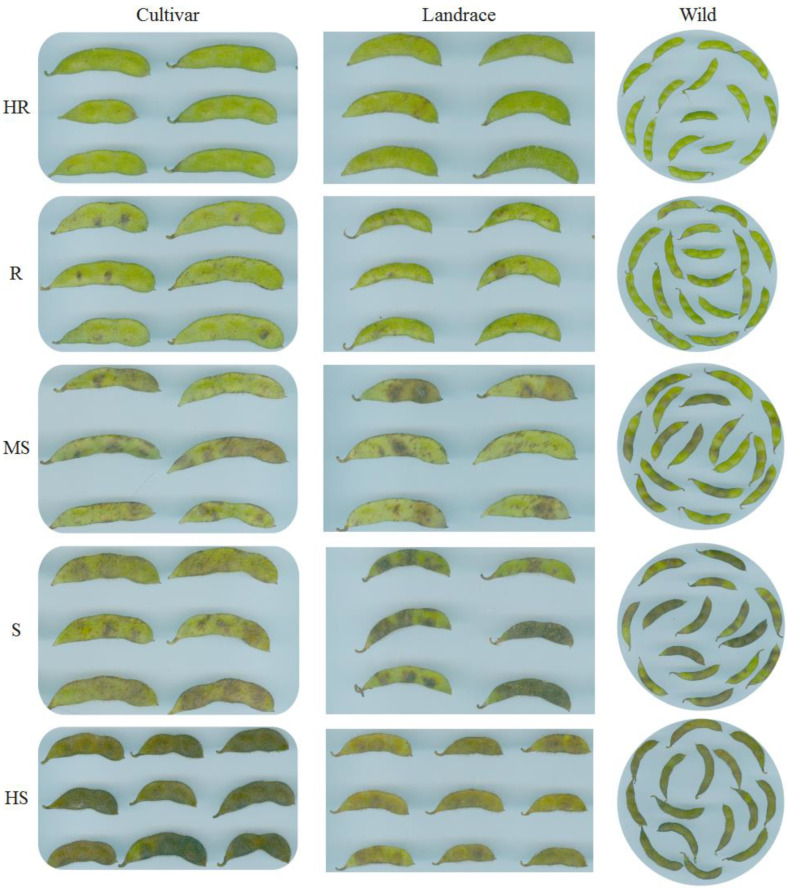
Representative symptoms for each resistance level and each germplasm resource type. HR, highly resistant; R, resistant; MS, moderately susceptible; S, susceptible; HS, highly susceptible.

The homogeneity of variance for DI values among the germplasm resources was significant (*p* < 0.001), indicating that the resistance levels of soybean germplasm resources were effectively distinguished. The resistance levels of all 589 germplasm resources nearly formed a normal distribution. Our screening revealed 37 HR (25 cultivars (C), 11 landraces (L), and 1 wild (W)), 148 R (100 C, 43 L, and 5 W), 210 MS (116 C, 77 L, and 17 W), 91 S (23 C, 52 L, and 16 W), and 103 HS (11 C, 50 L, and 42 W) individuals, but no germplasm resources exhibited immunity to anthracnose (Figure 6, Supplementary Table S1). The representative symptoms for each resistance level are shown in Figure 7.

**Figure 8:**
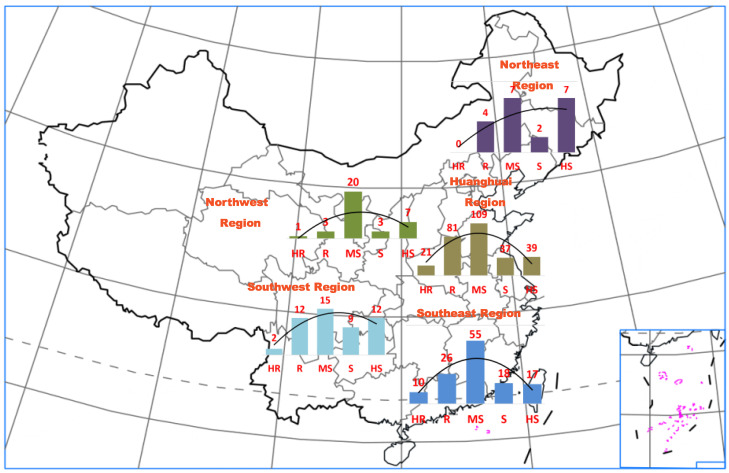
Geographic distribution of the soybean germplasm resources and the levels of anthracnose resistance. HR, highly resistant; R, resistant; MS, moderately susceptible; S, susceptible; HS, highly susceptible.

Among cultivars, the proportion of HR and R materials (125; 45.45%) was much larger than that of S and HS accessions (34; 12.36%). In contrast, the proportion of HR and R accessions (6; 7.41%) among wild materials was much smaller than that of wild S and HS accessions (58; 71.60%), and only one wild HR germplasm resource was identified. Among landraces, the distribution of resistance levels was close to a normal distribution. The resistance level and habitat of each germplasm resource are presented in Figure 8 and Supplementary Table S2. The resistance levels of the germplasm resources from the Huanghuai region and the region south of the Yangtze River were nearly normally distributed. Most germplasm resources from the northeast, northwest, and southwest regions were susceptible to soybean anthracnose, and only a few resistant germplasms have been identified. 

## 3. Discussion

The economic losses attributable to soybean anthracnose are significant, especially for vegetable soybean production. In addition to reducing yield, infection of fresh pods (the final product of vegetable soybean) with anthracnose also causes the commerciality (appearance and edibility) to decline, resulting in further economic loss. Therefore, the evaluation of the resistance of soybean germplasms, especially their pods, to anthracnose, and the screening of resistance resources is of great significance for the healthy and sustainable development of vegetable soybean production. 

In this study, we developed a rapid, stable, accurate, and safe method for the evaluation of soybean pods’ resistance to anthracnose. Unlike previous methods, based on in vivo inoculation and incubation under controlled laboratory conditions [14,16,17,18,19,20], our method involves in vitro inoculation and incubation under controlled laboratory conditions. The advantages of in vitro inoculation and incubation are as follows: stability and accuracy (fewer environmental effects), speed and efficiency (the ability to conduct multiple, repeated large-scale resistance evaluations), and safety (it will not cause pathogen accumulation in the test field). In vitro inoculation is not a novel method. Given the above-mentioned advantages, in vitro inoculation has been widely used in large-scale evaluations of plant resistance, and studies of the molecular mechanisms against diseases, such as rice blast [23], *Marssonina* apple blotch [24], wheat *Fusarium* head blight [25], and apple *Alternaria* blotch [26]. 

For soybean production, especially for vegetable soybean production, pod anthracnose is the most harmful, therefore, we chose to inoculate pods. Our results corroborate the finding that pods, consistent with other soybean plant tissues, are more susceptible to anthracnose when young [14,20]. However, the use of very young pods is not appropriate for large-scale resistance evaluations, because the shorter post-inoculation incubation period would reduce the time available for scanning blotchy areas without introducing large errors. Furthermore, sampling pods of the same maturity stage consistently is important; otherwise, large errors may be introduced. Type-I pods were thus, not suitable for sampling. The disease progression of type-II pods was much slower than that of type-I pods but slightly faster than those of type-III and type-IV pods. In addition, the visual and tactile characteristics of type-II pods were obvious, thus, improving sampling efficiency and reducing errors.

Another outstanding feature of our proposed method is the administration of inoculum by soaking instead of spraying, which saves time and improves efficiency. We found that both the inoculation methods gave highly consistent results in terms of disease progression and severity, demonstrating that either application method allows the mycelia to fully adhere to the pods. However, although suitable for in vitro inoculation, inoculation by soaking may not be applicable for in vivo use. 

The method developed in this study creates favorable conditions for the occurrence of anthracnose and establishes a strong screening pressure; consequently, this approach is beneficial for distinguishing differences in the resistance of different germplasm resources and for identifying materials with superior resistance. Although offering many advantages, our method is limited to the planting season. To fully exploit the available time and space, the exploration of methods that are completely laboratory-based, such as the use of soybean seedlings, is required. 

In this study, we used our proposed method to evaluate the anthracnose resistance of 589 soybean germplasm resources. The overall distribution of resistance levels was close to normal, which indicated that our method is feasible. We identified many resistant cultivars that can be applied for the resistance improvement of vegetable soybeans. As high-yield soybean cultivars, which are relatively few and genetically similar, dominate the production of autogamous soybean, the genetic diversity of modern soybean cultivars has declined to an alarmingly low level [27,28]. Although many resistant cultivars are known, their resistance may be due to only a few resistance genes. Many rare alleles that are likely to benefit future soybean improvement are present in wild and Asian landraces [29]. To our surprise, however, only a few of the landraces and wild accessions in our study exhibited resistance. In the future, the discovery of more resistance genes will, therefore, require the resistance evaluation of landraces and wild germplasm resources on an expanded scale.

## 4. Materials and Methods

### 4.1. Soybean Planting and Pods Sampling 

A total of 589 (275 C, 233 L, and 81 W) soybean germplasm resources, collected from across the Huanghuai region and the region south of the Yangtze River [22], were evaluated for anthracnose resistance; of these ZX9 and NBB were also used to develop the method for the evaluation of soybean anthracnose resistance. ZX9, a vegetable soybean cultivar, and NBB, a traditional soybean cultivar, are susceptible to anthracnose during cultivation. All soybean germplasm used in this study was planted at the experimental farm of the Zhejiang Academy of Agricultural Science, Jiaxing, China. To minimize the risk of soybean diseases and insect pests, we selected a field where legumes had not been planted for 8 years, for planting our soybean germplasms. Each germplasm resource was planted in three 1.3 m × 2.0 m plots, with inter-row spacings of 0.4 m and within-row plant spacings of 0.3 m.

Pods from healthy soybean plants in the field were collected and quickly placed in an incubator. Ice packs were used to maintain the temperature within the incubator at about 4 °C. The pods were separated from the ice packs with absorbable cotton to protect the pods from freezing injury. The sampled pods were transferred to the laboratory and washed with sterile water.

### 4.2. Fungal Isolate and Inoculum Preparation 

The fungal isolate CT5, a representative pathogen of anthracnose in vegetable soybean production, was obtained from infected pods of the vegetable soybean cultivar ZX9, collected in Longyou, Zhejiang Province, China (28.91797° N, 119.220583° E). The isolate was identified as *C. truncatum* based on its morphological characteristics and DNA sequence (GenBank accession: MW301345) [15]. The isolate was cultivated on potato dextrose agar (PDA) at 25 °C in darkness. 

Previous study has shown that mycelial suspensions of *C. truncatum* has several advantages over conidial suspensions as inoculums: mycelial suspensions are highly infectious, easy to prepare in large quantities, and time-saving [14]. The mycelial suspension used as the inoculum was prepared from isolate CT5 as follows. Ten 5 mm-diameter mycelial disks, from an actively growing culture of isolate CT5 on PDA, were added to 200 mL of sterilized potato dextrose broth (PDB) in 500 mL flasks. Each mycelial solution was incubated for 4 days in an incubator with shaking at 120 rpm, at 25 °C in darkness, and then filtered through sterilized gauze. The mycelial pellets were rinsed with sterilized water at least six times to remove as much residual PDB as possible, squeezed to minimize the water content, and weighed. The pellets were resuspended in sterilized water, fragmented in a blender (JR05A-300, Supor Household Products Co., Ltd., Hangzhou, China) at low speed for 20 s, and diluted with sterilized water to the required final concentration (50 mg mL^−1^). All steps were completed on an ultra-clean workbench. 

### 4.3. Determination of the Optimal Mycelial Suspension Concentration

Pods at the same stage of maturity, sampled from ZX9 and NBB, were inoculated with five concentrations of mycelial suspension (20, 30, 40, 50, and 60 mg mL^−1^) as follows. The pods were rinsed with sterilized water to remove debris and then inoculated with mycelial suspension by spraying with a hand-held watering can until the liquid ran-off. Inoculated pods were transferred onto filter papers in 15 cm Petri dishes containing 3 mL of sterilized water. The Petri dishes were then placed in an incubator at 25 °C under 14 h dark/10 h light conditions. Two to six DPI, the area covered by disease blotches was continuously scanned and calculated with a LA-S plant analysis system (Wseen, Shenzhen, China). Three repetitions were performed, and each repetition comprised 15 pods. 

### 4.4. Determination of the Optimal Pod Sampling Period 

Pods were sampled from ZX9 and NBB at four stages: type-I ca. 5 DAF, when the pods were small and soft; type-II ca. 15 DAF, when the pods were fully grown and no longer soft, but still flat because the seeds had not yet begun to protrude; type-III ca. 25 DAF, when the pods were half filled; and type-IV ca. 40 DAF, when the pods were completely filled, which is when vegetable soybean pods are harvested. The sampled pods were inoculated by spraying with a mycelial suspension (50 mg mL^−^^1^). The other inoculation and incubation steps were the same as those described above. Three repetitions were performed, and each repetition comprised 15 pods.

### 4.5. Determination of the Optimal Post-Inoculation Incubation Duration

The pods (type-II) sampled from 86 soybean germplasm resources were sprayed with a mycelial suspension (50 mg mL^−^^1^), prepared from mycelium incubated for 5 days. After 4, 5, and 6 days of incubation, blotchy regions were scanned, and their areas were calculated. All other inoculation and incubation steps were performed as described above.

Disease severity was scored on a scale of 0 to 5 based on the proportion of the pod surface covered by blotches as follows: 0, no visible blotches; 1 to 5, blotches covering 1.0% to 10.0% (1), 10.1% to 35.0% (2), 35.1% to 65.0% (3), 65.1% to 90.0% (4), and 90.1% to 100% (5) of the pods. The anthracnose resistance level of each germplasm resource was defined according to its calculated disease index (DI), i.e., {[(n_1_ × 1) + (n_2_ × 2) + (n_3_ × 3) + … + (n_n_ × n)/[N × (n_1_ + n_2_ + n_3_ + … + n_n_)]} × 100, where n_1_… n_n_ is the number of pods with a respective disease severity score, and N is the highest disease severity score. Six levels of anthracnose resistance were defined: immune (IM), DI = 0; HR, DI = 0.01 to 15.00; R, DI = 15.01 to 35.00; MS, DI = 35.01 to 65.00; S, DI = 65.01 to 85.00; and HS, DI = 85.01 to 100.

### 4.6. Comparison of Spray vs. Soak Inoculations

We first tested the effect of soaking duration on disease severity. Type-II pods sampled from ZX9 were placed in a flask containing 500 mL of mycelial suspension, gently stirred for 5, 10, 15, 20, or 25 s, and removed. After draining the excess mycelial suspension, inoculated pods were transferred to filter paper in 15 cm Petri dishes containing 3 mL of sterilized water. The Petri dishes were then placed in an incubator at 25 °C under 14 h dark/10 h light conditions. Following 2 to 6 days of incubation, disease blotches were scanned, and their areas were calculated using the LA-S plant analysis system (Wseen, Shenzhen, China). 

Next, type-II pods sampled from 10 randomly selected soybean accessions were inoculated by spraying or by 10 s of soaking, as described above. The inoculated pods were transferred to filter paper in 15 cm Petri dishes containing 3 mL of sterilized water. The Petri dishes were then placed in an incubator at 25 °C under 14 h dark/10 h light conditions. Disease blotches were scanned, and their areas were calculated after 3 to 6 days of incubation using the LA-S plant analysis system (Wseen, Shenzhen, China). 

### 4.7. Evaluation of Soybean Germplasm Resources for Anthracnose Resistance 

The 589 soybean germplasm resources (275 C, 233 L, and 81 W) were planted at the experimental farm of the Zhejiang Academy of Agricultural Science in the summer of 2019. Given the large number of soybean germplasm resources, resistance evaluation could not be completed simultaneously; the duration of post-inoculation incubation may differ by several hours among multiple resistance evaluations. Thus, ZX9, which showed stable disease progression and severity in multiple experiments, was chosen as a control. Seeds of ZX9 were sown in stages at intervals of 5 days; seeds were sown five times in total. Each resistance evaluation included ZX9. The same disease severity for ZX9 was observed for all resistance evaluations.

Anthracnose resistance was evaluated in type-II pods inoculated with 50 mg mL^−^^1^ mycelial suspension, using our newly developed in vitro method. The mycelial suspension was prepared from mycelium incubated for 4 days, and the inoculation was performed by soaking for 10 s. All other inoculation, incubation, scanning, and calculation steps were the same as described above. Then, the inoculated pods were transferred to filter paper in 15 cm Petri dishes containing 3 mL of sterilized water. The Petri dishes were then placed in an incubator at 25 °C under 14 h dark/10 h light conditions. Disease blotches were scanned, and their areas were calculated after about 5 days of incubation using the LA-S plant analysis system (Wseen, Shenzhen, China). Three replicates were evaluated for each germplasm resource evaluation and each planting plot was treated as one replicate. 

### 4.8. Data Analysis

Data analyses were conducted using SPSS Statistics 22.0 software (IBM Corp., Armonk, NY, USA). The homogeneity of variance for the proportion of the pod surface covered by blotches or the DI values among repeated experiments was tested using Bartlett’s test with replications as random effects. All differences between pairs were considered significant at *p* ≤ 0.05 based on two-tailed *t*-tests. Pearson correlation coefficients were calculated to evaluate the relationship between soak and spray inoculations. 

## 5. Conclusions

This study has yielded a new, rapid, reliable, and safe method for soybean anthracnose resistance evaluation. We used this method to evaluate the resistance of 589 soybean germplasm resources and identified many resistant accessions. Our work will be of importance for future research on soybean anthracnose, including work on resistance gene discovery, the elucidation of molecular mechanisms of resistance, and resistance breeding. Although offering many advantages, our method is limited to the planting season. The exploration of completely laboratory-based methods, such as the use of soybean seedlings for resistance evaluation, is needed to maximize available time and space.

## Data Availability

All data generated or analysed during this study are included in this published article and its supplementary information files.

## Supplementary Materials

The following supporting information can be downloaded at: https://www.mdpi.com/article/10.3390/plants11050657/s1, Table S1: The resistance levels of 589 germplasm resources; Table S2: Geographic distribution of the soybean germplasm resources and the levels of anthracnose resistance.
